# Autoregulation of topoisomerase I expression by supercoiling sensitive transcription

**DOI:** 10.1093/nar/gkv1088

**Published:** 2015-10-22

**Authors:** Wareed Ahmed, Shruti Menon, Pullela V. D. N. B. Karthik, Valakunja Nagaraja

**Affiliations:** 1 Department of Microbiology and Cell Biology, Indian Institute of Science, Bangalore, India; 2 Jawaharlal Nehru Centre for Advanced Scientific Research, Bangalore, India

## Abstract

The opposing catalytic activities of topoisomerase I (TopoI/relaxase) and DNA gyrase (supercoiling enzyme) ensure homeostatic maintenance of bacterial chromosome supercoiling. Earlier studies in *Escherichia coli* suggested that the alteration in DNA supercoiling affects the DNA gyrase and TopoI expression. Although, the role of DNA elements around the promoters were proposed in regulation of gyrase, the molecular mechanism of supercoiling mediated control of TopoI expression is not yet understood. Here, we describe the regulation of TopoI expression from *Mycobacterium tuberculosis* and *Mycobacterium smegmatis* by a mechanism termed Supercoiling Sensitive Transcription (SST). In both the organisms, *topoI* promoter(s) exhibited reduced activity in response to chromosome relaxation suggesting that SST is intrinsic to *topoI* promoter(s). We elucidate the role of promoter architecture and high transcriptional activity of upstream genes in *topoI* regulation. Analysis of the promoter(s) revealed the presence of sub-optimal spacing between the −35 and −10 elements, rendering them supercoiling sensitive. Accordingly, upon chromosome relaxation, RNA polymerase occupancy was decreased on the *topoI* promoter region implicating the role of DNA topology in SST of *topoI*. We propose that negative supercoiling induced DNA twisting/writhing align the −35 and −10 elements to facilitate the optimal transcription of *topoI*.

## INTRODUCTION

Negative supercoiling of the bacterial chromosome is essential for the optimal functioning of the DNA transaction processes (1,2). Negative super-helical density, in general, facilitates the promoter melting and promotes the transcription of genes (3). Alteration in the steady state level of chromosome supercoiling influences the bacterial gene expression (4) and hence chromosome supercoiling has to be regulated for the optimal growth and physiology of the cell. DNA topoisomerases are the major regulators of chromosome supercoiling in bacteria (2). In *E. coli*, DNA gyrase introduces the negative supercoiling while topoisomerase I (TopoI) and topoisomerase IV (TopoIV) relax the excess supercoiling generated by DNA gyrase and active transcription/replication machinery (5–8). For the regulation of chromosome supercoiling, there must be a mechanism to sense, interpret and respond to alterations in super-helical density to bring it back to the steady-state level. Indeed, a mechanism has evolved for the regulation of topoisomerases which operates by sensing the alterations in the chromosome supercoiling (9–12). The response is generated in the form of alteration in the expression of topoisomerases. The regulation of topoisomerase expression has been studied in *E. coli* (12–14). Expression of the supercoiling enzyme DNA gyrase was shown to increase in response to relaxation (14). This phenomenon of autoregulation of DNA gyrase is termed as Relaxation Stimulated Transcription (RST) (10). On the other hand, expression of DNA TopoI—the primary relaxase in *E. coli* was found to increase marginally when chromosome was negatively supercoiled (9) and the expression was significantly down-regulated in response to chromosome relaxation (12). Such autoregulation of the expression of topoisomerases facilitates the maintenance of topological homeostasis in the cell.

The underlying mechanism for gyrase regulation has been elucidated in *E. coli* and mycobacteria. In *E. coli*, the regulatory mechanism of *gyrA* and *gyrB* expression is an attribute of the intrinsic property of DNA elements in and around the promoter, particularly the −10 region (10,15–17) while in *M. smegmatis* and *M. tuberculosis* the role of the distal promoter elements and overlapping promoter has been implicated in the regulation of the gyrase operon, respectively (18,19). Studies in *E. coli* identified the supercoiling responsive promoters of *topoI* (11,12). The promoter(s) activity was found to alter with the change in environmental condition and the role of sigma factors in the regulation of *topoI* expression was deciphered (20,21). However, the molecular mechanism or the involvement of DNA elements in conferring the supercoiling sensitivity to *topoI* promoter(s) remains to be elucidated.

Several members of the genus *Mycobacterium* encounter unfavorable environments and adapt to hostile conditions (22,23). DNA supercoiling and topoisomerases may assist in the re-configuration of gene expression required for such adaptations (24). The mycobacterial chromosome encodes a single Type IA enzyme which has been shown to be essential for the cell growth (25). The absence of additional relaxases (unlike in *E. coli*) in mycobacteria suggests the additional responsibility for TopoI in regulating the relaxation of the chromosome. In order to sustain the optimal chromosome supercoiling, the topoisomerase activity in the mycobacterial chromosome needs to be appropriately balanced. We describe the regulation of *topoI* in non-pathogenic *M. smegmatis* and the pathogenic *M. tuberculosis*. Mapping of the transcripts of *topoI* in both the mycobacterial species showed the presence of two promoters. Both the promoters were found to be sensitive to the change in chromosome supercoiling and their intrinsic properties contribute in the Supercoiling Sensitive Transcription (SST) of *topoI* in both the organisms. In addition high transcription of an upstream gene affected the topology of *topoI* regulatory region, influencing its activity.

## MATERIALS AND METHODS

### Bacterial strains, growth media and transformation conditions

The following bacterial strains were used: *E. coli* DH10B (laboratory stock), *M. smegmatis* mc^2^ 155 (laboratory stock), *M. tuberculosis* H37Ra. *E. coli* strains were grown at 37°C in Luria–Bertani (LB) broth or on LB agar plates. Mycobacterial strains were grown in Middlebrook 7H9 broth (Difco) or 7H10 agar plates (Difco), supplemented with 0.2% glycerol and 0.05% Tween-80 at 37°C. For the *M. tuberculosis*, the medium was supplemented with 10% ADC (Albumin, Dextrose and NaCl). Antibiotics were added to the media at the following concentrations: 25 μg/ml Kanamycin (Sigma Aldrich, CA, USA), 25 ng/ml Tetracycline (Sigma Aldrich, CA, USA).

### Cloning of *topoI* gene and its promoter

TopoI overexpressing constructs were generated in pMIND vector system (26). The *M. smegmatis topoI* gene was amplified from pPVN123 (27). The polymerase chain reaction (PCR) products were digested with NdeI and EcoRV and cloned into pMIND vector linearized with NdeI and EcoRV (26). Clones were confirmed by double digestion with NdeI and BamHI, and the expression of TopoI in *M. smegmatis* cells was monitored by immunoblotting. The 1.5 kb upstream promoter regions of *M. smegmatis topoI* and *M. tuberculosis topoI* were cloned upstream to the β-galactosidase gene in the pSD5B promoterless vector (28) at the XbaI site. This construct (2 μg plasmid) was electroporated into *M. smegmatis*. Recombinant colonies were selected on 7H10 agar plates containing Kanamycin (25 μg/ml). For the insertion of nucleotides in the spacer region of PMstopo2, megaprimer inverse PCR mutagenesis strategy was employed (29). Briefly, the 530 bp upstream region of *topoI* gene cloned into the pSD5B was used as a template and forward primers containing 3 or 4 additional nucleotides were utilized to introduce insertion mutations in the spacer of major promoter (based on expression) Mstopo2.

### Immunoblot analysis

25 μg of total cell lysates were separated on 8% sodium dodecyl sulphate-polyacrylamide gel electrophoresis and transferred to PVDF membranes. Prior to probing with antibody, the equal loading and transfer of lysates to membrane was ensured by Ponceau S staining. Membranes were incubated in PBS blocking buffer (10 mM Na- phosphate, pH 7.5, 150 mM NaCl, 0.05% Tween 20) with 2% (w/v) BSA for 2 h prior to incubation with primary antibodies diluted (1:20 000) in PBS with 2% BSA for 2 h. Membranes were washed in PBST (.05% Tween 20) three times, and then incubated with secondary antibodies for 2 h followed by washing three times with PBST. Protein bands were visualized using chemiluminescent substrates (Millipore).

### RNA extraction and qPCR

RNA was extracted from *M. smegmatis* and *M. tuberculosis* exponentially grown cells using a Qiagen RNeasy kit following the manufacturer's protocol. From the total RNA, cDNAs were synthesized using a high-capacity cDNA reverse transcription kit (Applied Biosystems). cDNA generated with random primers was used for quantitative real-time PCR (qPCR), with SYBR green as the indicator dye. The expression of the genes was quantified after normalization of RNA levels to the expression of the *sigA* transcript. The qPCR cycling conditions were as follows: 95°C for 2 min, followed by 40 cycles of 95°C for 15 s, 57°C for 30 s and 72°C for 20 s.

### Primer extension

For primer extension, 5 μg of total RNA isolated from log phase cultures of *M. smegmatis* or *M. tuberculosis* was heated to 65°C, rapidly chilled, followed by the addition of primers (which anneals to the 5′end of *topoI* coding region) and incubation at 55°C, again chilled and extension mix (containing 10 mM dNTP mix and 200 units RevertAid Premium Reverse Transcriptase) was added followed by incubation at 50°C for 1 h. The products were separated in a 6% sequencing gel. The size of the products was deduced using a sequencing reaction. For sequencing, the pUC-T7A1 plasmid and pUC forward primer were used. Gels were scanned in a Typhoon 9500 (GE) phosphorimager.

### β-galactosidase reporter assay

Promoter strength was measured by β-galactosidase reporter assay and the activity represented in Miller units [(Miller units = 1000 × *A*_420/_ (time (min) x volume of culture (ml) x optical density at 600 nm)](30). *M. smegmatis* harboring vector pSD5B was used as the negative control. To determine the *in vivo* promoter strength in different growth phases, promoter fusion constructs were introduced into *M. smegmatis* and the resulting strains were grown for 4, 8, 12, 16, 26, 32, 40 h and promoter activity was determined.

### Chromatin immunoprecipitation (ChIP) and RT PCR

ChIP with exponentially grown *M. smegmatis* cultures was carried out as described previously (31). Briefly, formaldehyde cross-linked cells were lysed by sonication (by Bioruptor; Diagenode) to shear the DNA. The fragmented DNA was immunoprecipitated by using the anti-RpoB or anti-GyrA antibody and purified. The resulting ChIP-DNA was subjected to qRT PCR analysis to determine the enrichment of the *topoI* promoter region (or other targets) in immunoprecipitated (IP) sample over the mock-IP (without antibody) sample.

## RESULTS

### Supercoiling sensitive expression of TopoI

**Figure 1.**
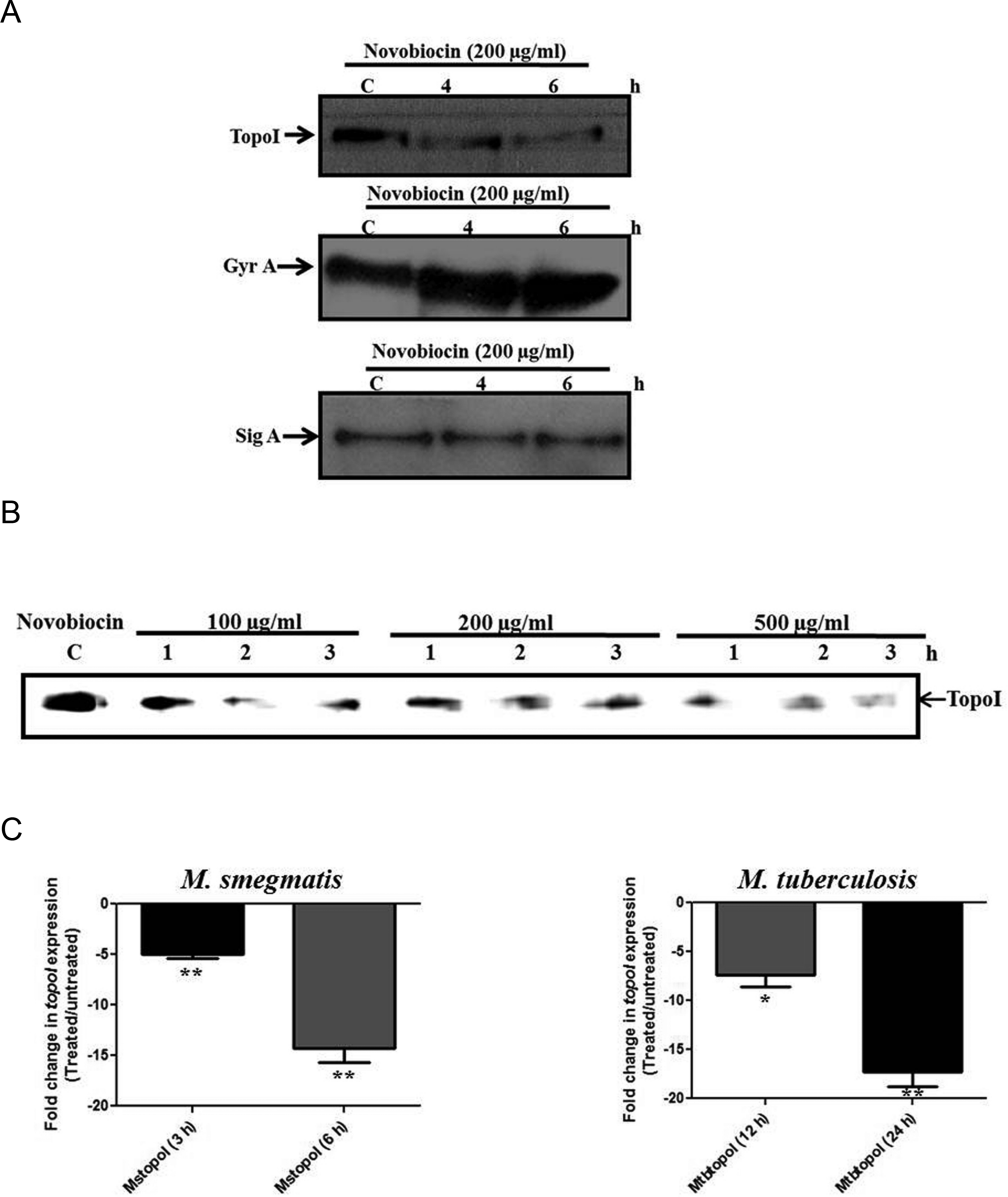
TopoI expression is sensitive to gyrase inhibition. *M. smegmatis* cells were grown to exponential phase and treated with the different concentrations of novobiocin to induce chromosome relaxation. The cells were lysed and subjected to immunoblot analysis using specific antibodies against the proteins of interest (as indicated in the panel). (**A**) *M. smegmatis* cells treated with 200 μg/ml novobiocin for 4–6 h and the expression of TopoI, GyrA and SigA was monitored. (**B**) Dose dependent repression of TopoI expression by novobiocin. The *M. smegmatis* cells were exposed to various concentrations of novobiocin for different time and TopoI expression was monitored. (**C**) Transcript analysis of TopoI expression. The exponential phase cells of *M. smegmatis* and *M. tuberculosis* treated with the novobiocin (100 μg/ml) for 3 h–6 h and 12–24 h, respectively. Total RNA was isolated from treated and untreated cells and cDNA was prepared using random hexamer primers. Abundance of *topoI* transcript was measured by qRT PCR analysis using gene specific primers. Fold change in *topoI* expression compared to the untreated culture*. sigA* was used as a reference gene for the expression analysis. The error bars represent the SD (standard deviation) obtained from three independent experiments. *P < 0.01, **P < 0.001.

**Figure 2.**
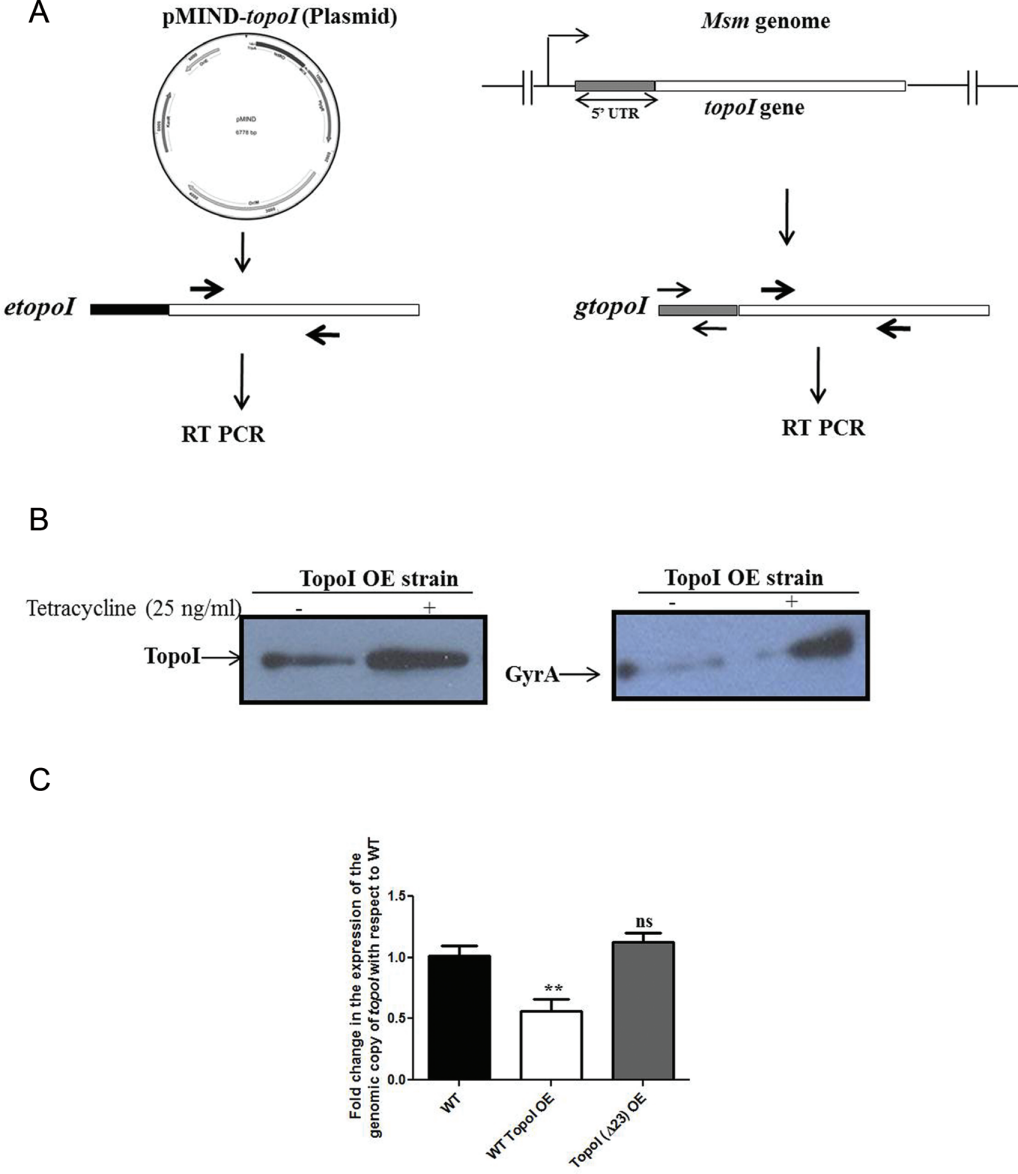
Reduced expression of TopoI upon chromosome relaxation. The chromosome relaxation was carried out by ectopic overexpression of *M. smegmatis* TopoI using tetracycline inducible system. The exponential phase *M. smegmatis* cells were treated with the tetracycline (25 ng/ml) for 6 h and the expression of TopoI and DNA gyrase was monitored both at protein as well as RNA level. (**A**) Schematic for the discriminatory qRT PCR. The 5′ UTR of the ectopically expressed *topoI (etopoI)* and genomic copy of *topoI* (*gtopoI*) were different. The primers specific for *gtopoI* were used to determine the alteration in the expression of *gtopoI* upon TopoI overexpression (to cause chromosome relaxation). (**B**) Immunoblot analysis of TopoI and gyrase expression upon induction with the tetracycline. The increased overexpression of GyrA upon TopoI overexpression indicates the chromosome relaxation (RST). (**C**) Measurement of the genomic *topoI* transcript abundance in the cells upon overexpression of TopoI by discriminatory qRT PCR analysis. The fold change is expression represents the expression of *topoI* transcripts upon tetracycline induction normalized with the uninduced RNA samples. topoI (ORF) indicates the expression of total *topoI* transcripts; *gtopoI* and *etopoI* represents the expression of genomic copy and plasmid copy of *topoI* transcripts, respectively. The error bars represents the SD obtained from three independent experiments. *P < 0.01, **P < 0.001, ns: not significant (*P* > 0.1).

In order to decipher the effect of different cues on the expression of TopoI, *M. smegmatis* cells were exposed to different stress conditions and TopoI expression was monitored. Out of the several conditions tested, the DNA gyrase inhibitor—novobiocin, drastically affected the expression of TopoI (Supplementary Figure S1). The inhibition of DNA gyrase induces the relaxation of the chromosome (32,33) and thus the observed reduction in the TopoI level in the presence of novobiocin suggested the supercoiling sensitive expression of TopoI. The analysis of mycobacterial cell lysates prepared from the novobiocin treated cells showed an enhanced expression of DNA gyrase indicating the operation of relaxation stimulated transcription (RST) of DNA gyrase in mycobacteria (Figure 1A and Supplementary Figure S2). Further, with an increase in the novobiocin concentration or the time of exposure, the level of TopoI was found to decrease gradually (Figure 1B and Supplementary Figure S2). The transcript analysis of *topoI* suggested the decrease in the transcript abundance of the gene (Figure 1C) suggesting that the *topoI* gene exhibits SST. The validation of the SST of *topoI* was carried out by the relaxation of the chromosome of *M. smegmatis* by overexpressing the TopoI under tetracycline inducible system and scoring the transcription of genomic copy of *topoI*. The unique 5′UTR of the genomic copy of *topoI* forms the basis of discriminatory qRT PCR allowing the measurement of alteration in the transcript abundance of the genomic copy of *topoI* even in the presence of plasmid copy of *topoI* (Figure 2A). To ensure that the overexpression of TopoI upon induction with tetracycline led to the chromosome relaxation plasmid DNA were isolated and their topology was analyzed by chloroquine-agarose gel electrophoresis. Indeed, the plasmid isolated from the cells overexpressing TopoI exhibits accumulation of the relaxed topoisomers (Supplementary Figure S3). Moreover, the overexpression of TopoI led to the increased expression of DNA gyrase which is an indication of RST—a characteristic of chromosome relaxation (10) (Figure 2B). The discriminatory RT PCR analysis revealed the concomitant decrease in the abundance of transcript of the genomic copy of *topoI* (Figure 2C). To confirm that the reduction in the genomic copy of *topoI* was an attribute of the chromosome relaxation brought about by TopoI and not the result of overproduction of TopoI, we overexpressed TopoI mutant which is relaxation deficient (TopoIΔ23) (34). The overexpression of the mutant did not affect the expression of chromosomal *topoI* suggesting that DNA relaxation induced by TopoI down-regulates its own expression (Figure 2C). Overall, the data confirm the supercoiling sensitive autoregulation of *topoI* in mycobacteria.

### Evaluation of mycobacterial *topoI* promoter activity

To assess the activity of mycobacterial *topoI* promoter(s), the 1.5 kb region encompassing the two upstream genes (MSMEG_6158/Rv3647c and MSMEG_6159/Rv3648c) and putative *topoI* promoters (Supplementary Figure S4) were cloned in the promoter less plasmid pSD5B (28), harboring the reporter gene β-galactosidase for the promoter activity analysis. The constructs were introduced into both *E. coli* and *M. smegmatis* cells to monitor the expression of reporter gene cloned under the *topoI* promoter(s). The expression of β-galactosidase was not seen in the *E. coli* while the promoter activity was observed in *M. smegmatis* (blue colonies, Supplementary Figures S5A and S6A) suggesting that the expression of *topoI* promoter(s) was restricted to mycobacteria. The inability of *topoI* promoter to express in *E. coli* could be associated with the differences in the promoter recognition motifs/elements between the two organisms. The activity of both the *M. tuberculosis* and *M. smegmatis topoI* promoters was found to be growth-phase dependent (Supplementary Figures S5B and S6B). In early exponential phase, the expression was found to be highest which decreased with the rise in the culture's optical density.

Since, the *topoI* exhibits supercoiling sensitive expression, we monitored the influence of the environmental conditions which are known to alter the DNA topology. An increase in extracellular osmolarity was shown to elevate the *in vivo* DNA supercoiling in *E. coli* and *Salmonella* (35,36). Similarly, the high salt concentration or hypertonic sucrose solution led to an enhancement in the mycobacterial *topoI* promoter activity indicating the supercoiling induced activity of *topoI* promoter(s) (Supplementary Figure S7A and S7B). The change in the growth temperature is also known to affect the plasmid linking number/DNA topology (37) although the direction of the alteration varies across the bacterial species (37–39). The increase in the relaxed topoisomers of the plasmid isolated from the *M. smegmatis* growing at 42°C suggested the relaxation of the chromosome (Supplementary Figure S7C). These alterations in DNA topology are similar to what is seen in *Shigella*, *Salmonella* and halophilic archaeon *Haloferax volcanii* (38,39) but opposite to that of *E. coli* (37). The temperature dependent decrease in *topoI* promoter activity seen for mycobacterial promoter is the consequence of DNA relaxation (Supplementary Figure S6C). Moreover, the activity of the *topoI* promoters in the plasmid context was also found to be reduced in response to the DNA relaxation achieved by gyrase inhibition (Supplementary Figures S5C and S6C), suggesting the involvement of promoter elements in such supercoiling sensitive regulation. To sum up, the activity of the *topoI* promoters is dynamically regulated and responsive to the alterations in the environmental conditions.

### Upstream region of *topoI* promoter influences its activity

**Figure 3.**
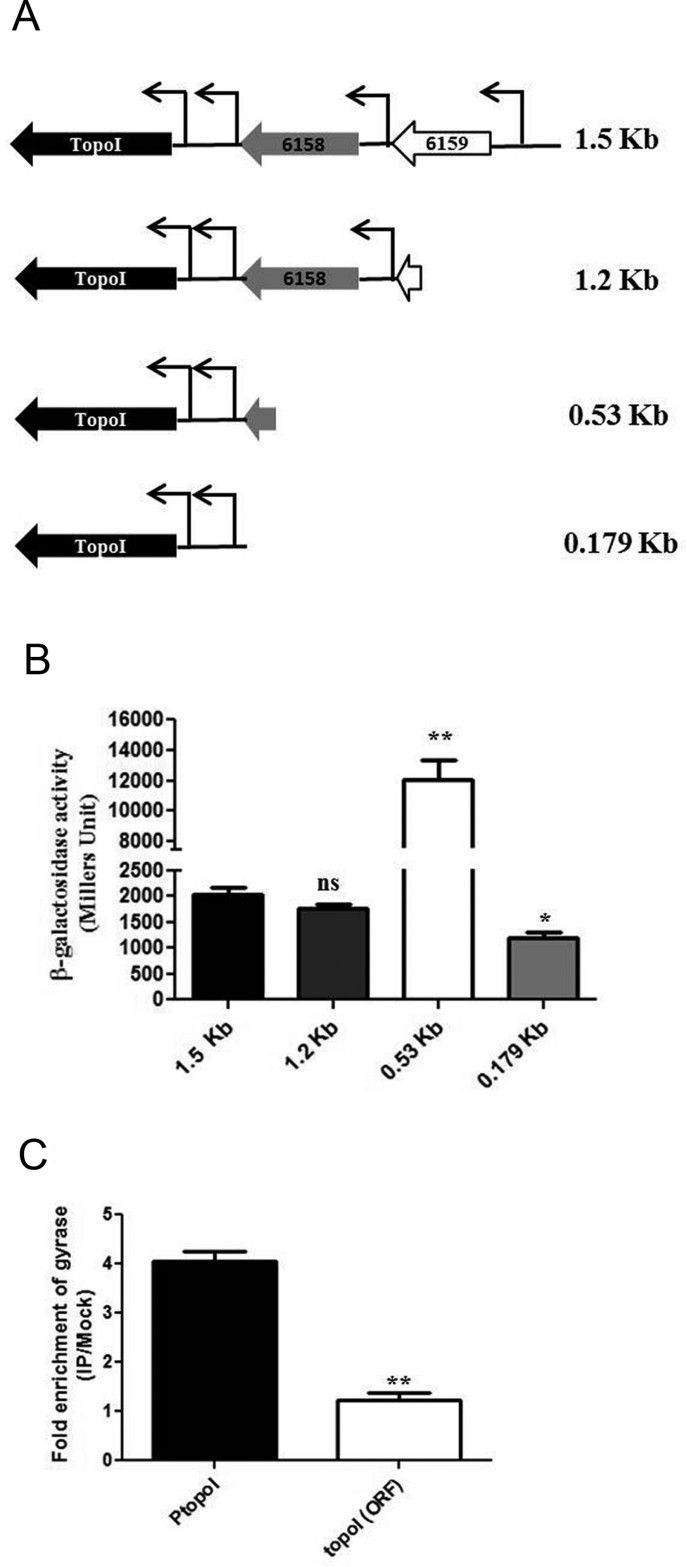
Contribution of upstream elements on *topoI* promoter activity. (**A**) Schematic representation of the constructs generated for the study. (**B**) Measurement of the activity of various constructs harboring *topoI* promoter(s) by β-galactosidase assay. (**C**) Determination of gyrase binding on upstream region of *topoI* by ChIP-qRT PCR using the primers specific to the *topoI* upstream region. The enrichment values represent the enrichment of DNA fragment of interest (*topoI* promoter region and ORF) in immunoprecipitated (IP) sample over the mock. The error bars represent the SD obtained from three independent experiments.*P < 0.01, **P < 0.001, ***P < 0.0001, ns: not significant (*P* > 0.1).

Analysis of the arrangement of the *topoI* gene and neighboring genes in the chromosomes of various mycobacterial species revealed a conserved pattern (Supplementary Figure S4). Importantly, the arrangement of upstream genes was highly conserved. One explanation for the retention of the same set of genes could be their role in the regulation of expression of the downstream genes. To monitor the effect of these genes on the *topoI* promoter activity, various deletion constructs of *topoI* upstream region were generated (Figure 3A), cloned into pSD5B reporter plasmid and their activity was evaluated by the β-galactosidase assay (Figure 3B). Deletion of a part of the immediate upstream gene MSMEG_6158 with its entire regulatory region resulted in ≈10-fold higher *topoI* promoter activity (530 bp construct) compared to the 1.5 kb construct retaining the intact MSMEG_6158 gene. However, the deletion of the next upstream gene MSMEG_6159 alone (1.2 kb construct) did not reveal significant change in the *topoI* promoter expression. The expression analysis of MSMEG_6158 indicated the high abundance of its transcripts (Supplementary Figure S8). Similarly, expression data from *M. tuberculosis* showed high transcription activity of Rv3647c which is a homolog of MSMEG_6158, located in the identical position in the *M. tuberculosis* chromosome (31). Analysis of MSMEG_6158 sequence did not reveal any known motifs for DNA binding or features of activator/repressor proteins. The higher transcriptional activity of the MSMEG_6158 could potentially be influencing the topology of the regulatory region of *topoI* leading to its reduced expression. Notably, the further deletion of the *topoI* upstream region led to the drastic reduction in the promoter activity (0.179 kb construct) (Figure 3B).

### *topoI* upstream region recruits DNA gyrase

Higher transcriptional activity of the upstream gene of *topoI* promoter could result in the accumulation of positive supercoils (40,41) at the end of the MSMEG_6158 transcription unit located ahead of *topoI* thus perturbing the topology of the *topoI* regulatory region leading to the reduced expression. Negative supercoiling facilitates the *topoI* expression while the DNA relaxation has the opposite effect. Thus the optimal expression of the *topoI* gene requires the maintenance of a negative supercoiled state in the promoter and the neighboring sequences. To evaluate the role of gyrase in the stimulation of *topoI* expression as it is primarily responsible for introduction of negative supercoils in the chromosome, the binding of DNA gyrase in the regulatory region of *topoI* was assessed by ChIP-qRT PCR. High occupancy of DNA gyrase on the regulatory region of *topoI* and low occupancy on the gene ORF suggests the specific recruitment of DNA gyrase (Figure 3C). To identify the gyrase binding sites in the *topoI* upstream region, DNA bound gyrase was trapped with Moxifloxacin (Moxi) and ternary complexes were immunoprecipitated. The region between the 530 bp and 179 bp fragment of *topoI* upstream region recruits DNA gyrase and deletion of it abrogates the gyrase binding (Supplementary Figure S9A). Further, the reduced gyrase binding on the upstream region of 0.179 kb construct correlates with the significant drop in its promoter activity (Figure 3B).

To establish that DNA gyrase recruited at the *topoI* promoter is involved in the introduction of negative supercoiling in the promoter region, we have carried out biotinylated psoralen (bpsoralen) crosslinking was carried out as described (42). Psoralen preferentially intercalates into the negatively supercoiled DNA and thus the enhanced association of psoralen is an indicator of underwound DNA. When the gyrase activity was inhibited enrichment of bpsoralen-associated *topoI* promoter DNA was reduced (Supplementary Figure S9B) indicating the direct role of gyrase binding in the introduction of negative supercoils at *topoI* promoter(s). Overall, the gyrase recruitment at *topoI* regulatory regions appears to be essential for the maintenance of negative supercoiled status and hence the activity of its promoter.

### Mapping of transcription start site (TSS) for *topoI* from *M. smegmatis* and *M. tuberculosis*

**Figure 4.**
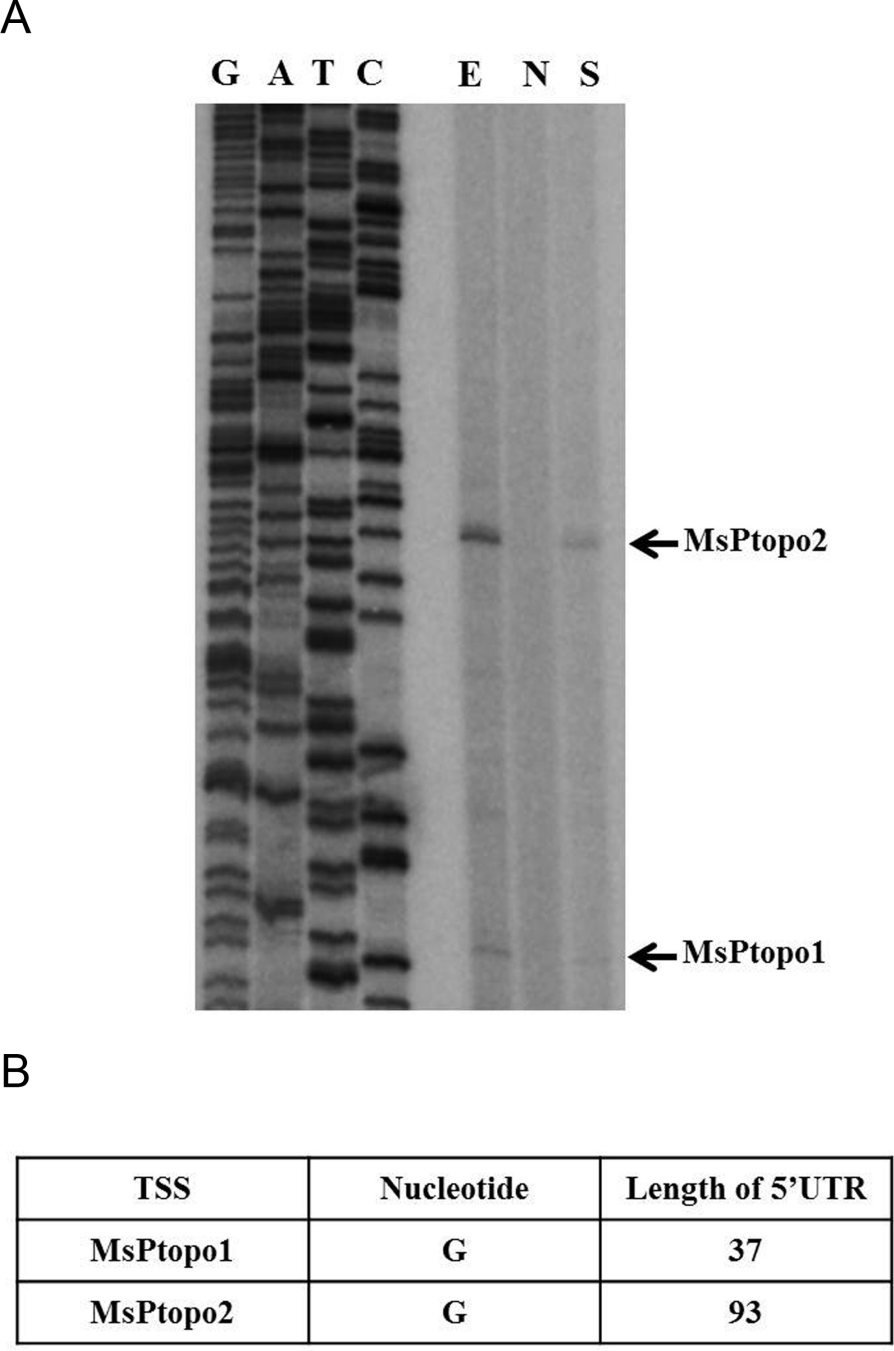
Identification of Transcription Start Sites of *topoI* gene from *M. smegmatis*. (**A**) Primer extension analysis was carried out to map the TSS upstream of *topoI* as described in Materials and Methods. The primer extension products corresponding to the transcription start site for each promoter is indicated (arrows). E: exponential phase culture without novobiocin treatment, N: exponential phase culture treated novobiocin (100 μg/ml) for 3 h, S: stationary phase culture without novobiocin. (**B**) Table representing the 5′ UTR length and first nucleotide of the each transcripts.

The operation of SST of *topoI* raised a question on the regulatory mechanism and underlying elements for such regulation. In order to address this, mapping of TSS for the *topoI* mRNA from *M. smegmatis* and *M. tuberculosis* was carried out. The RNA from the mycobacterial cells was subjected to primer extension and the size of the extension products was analyzed to map the TSS. Two extension products were identified in both *M. smegmatis* and *M. tuberculosis*, indicating the existence of two promoters for the *topoI* transcription (Figure 4A and Supplementary Figure S10). Analysis of the *topoI* transcripts revealed the presence of longer 5′ leader sequences (Figure 4B and Supplementary Figure S10B). Notably, all the *topoI* transcripts, from both *M. smegmatis* and *M. tuberculosis*, were significantly reduced upon novobiocin exposure suggesting the supercoiling sensitive expression of all the transcripts.

### Supercoiling sensitive transcription of *topoI* is conferred by its native promoter(s)

**Figure 5.**
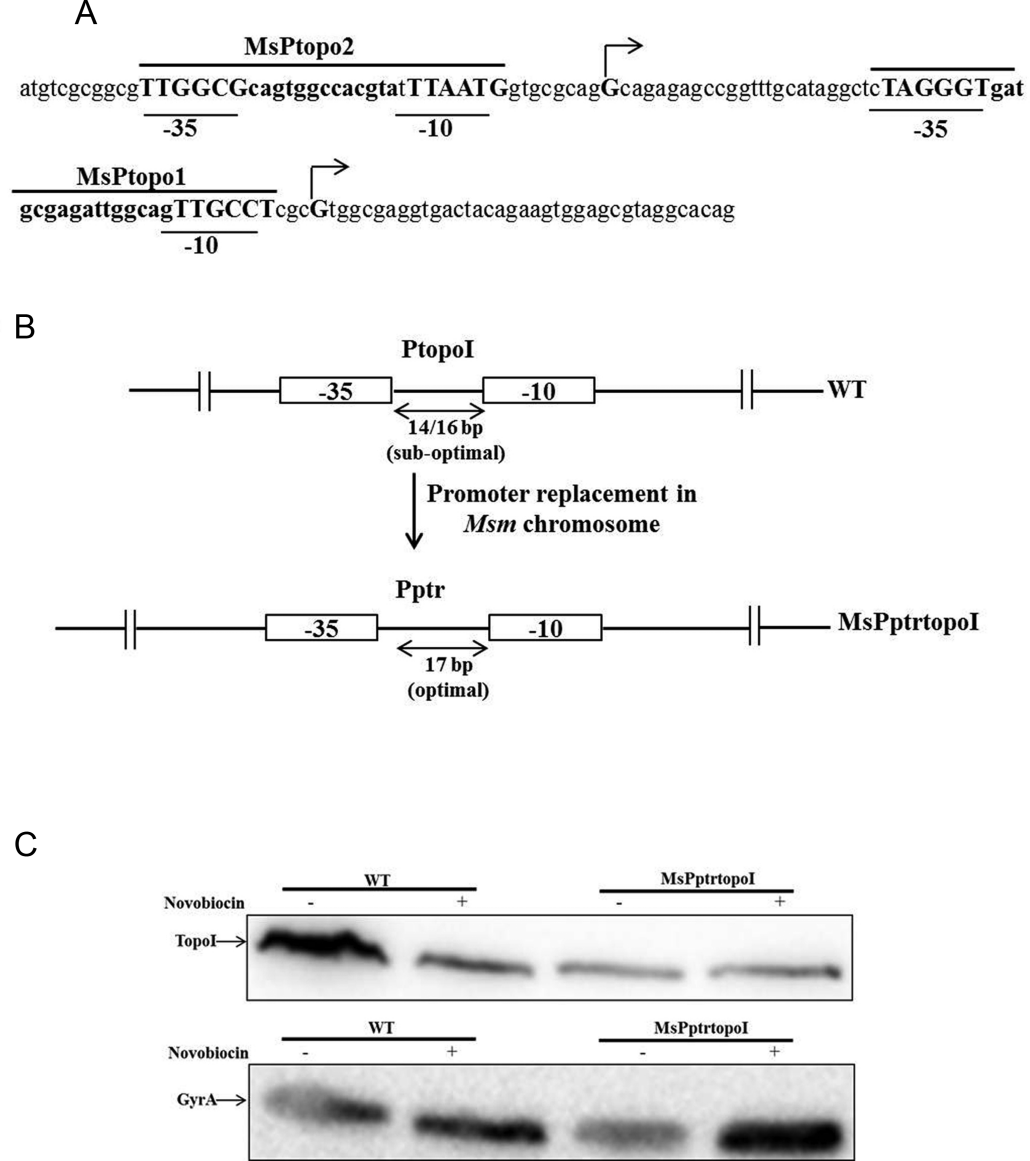
Supercoiling sensitive expression of *topoI* is specific to native *topoI* promoter(s). (**A**) Promoter elements upstream to the start sites. Arrows indicates the transcription start sites. (**B**) Schematic showing the replacement of native promoter(s) of *topoI* with a *ptr* promoter (having optimally spaced −35 and −10 elements—17 bp spacer). (**C**) Immunoblot analysis of the TopoI and gyrase expression in native *M. smegmatis* cells and mutant strain with *ptr* promoter. The exponential phase recombinant and WT *M. smegmatis* cells were treated with novobiocin (100 μg/ml) for 6 h and processed for the immuno-detection of TopoI.

The results described so far indicated that the *topoI* promoter and upstream region contribute to the supercoiling sensitive expression of *topoI*. In order to evaluate the specificity of the *topoI* promoter(s) for such regulation, a strain was used where the native promoter(s) of *topoI* was replaced with a *ptr* promoter (P*ptr*) (Schematic Figure 5B) (43). Wild type (WT) *M. smegmatis* cells and the recombinant strain were treated with novobiocin and the expression of topoisomerases was monitored by immunoblotting. In both the WT and recombinant strain, the expression of gyrase was found to be increased upon treatment with novobiocin indicating the relaxation of the chromosome. The WT cells showed a decrease in the TopoI expression while the recombinant strain did not show a significant change in the expression of TopoI (Figure 5C) indicating that only the native *topoI* promoter(s) exhibited such a supercoiling sensitive response.

To decipher the underlying mechanism and regulatory elements involved in the regulation of *topoI* gene expression, promoter sequences of *topoI* from mycobacteria were analyzed. The spacer length between the −35 and −10 elements was found to be 14–16 nucleotides (Figure 5A). Due to the helical nature of DNA, the length of the spacer between the promoter elements is crucial to orient them into the same phase (44). According to this ‘twist’ model, promoters with optimal spacer length would show constitutive expression while the suboptimal spacing between the −10 and −35 motifs influence their relative orientation conferring supercoiling sensitivity. Thus, we reasoned that *topoI* promoter(s) may be subjected to similar supercoiling sensitive regulation. To validate the hypothesis we introduced 3 and 4 nucleotides in the spacer region of the native *topoI* promoter (major promoter MsPtopo2) by site directed mutagenesis using the 530 bp PtopoI-pSD5B construct as a template. Upon chromosome relaxation, the mutated constructs having 17/18 bp spacers did not show the drastic reduction in the promoter activity unlike the native promoter (Supplementary Figure S11) confirming the role of promoter elements in SST of *topoI*.

### Chromosome relaxation affects the RNAP occupancy on the *topoI* promoter

**Figure 6.**
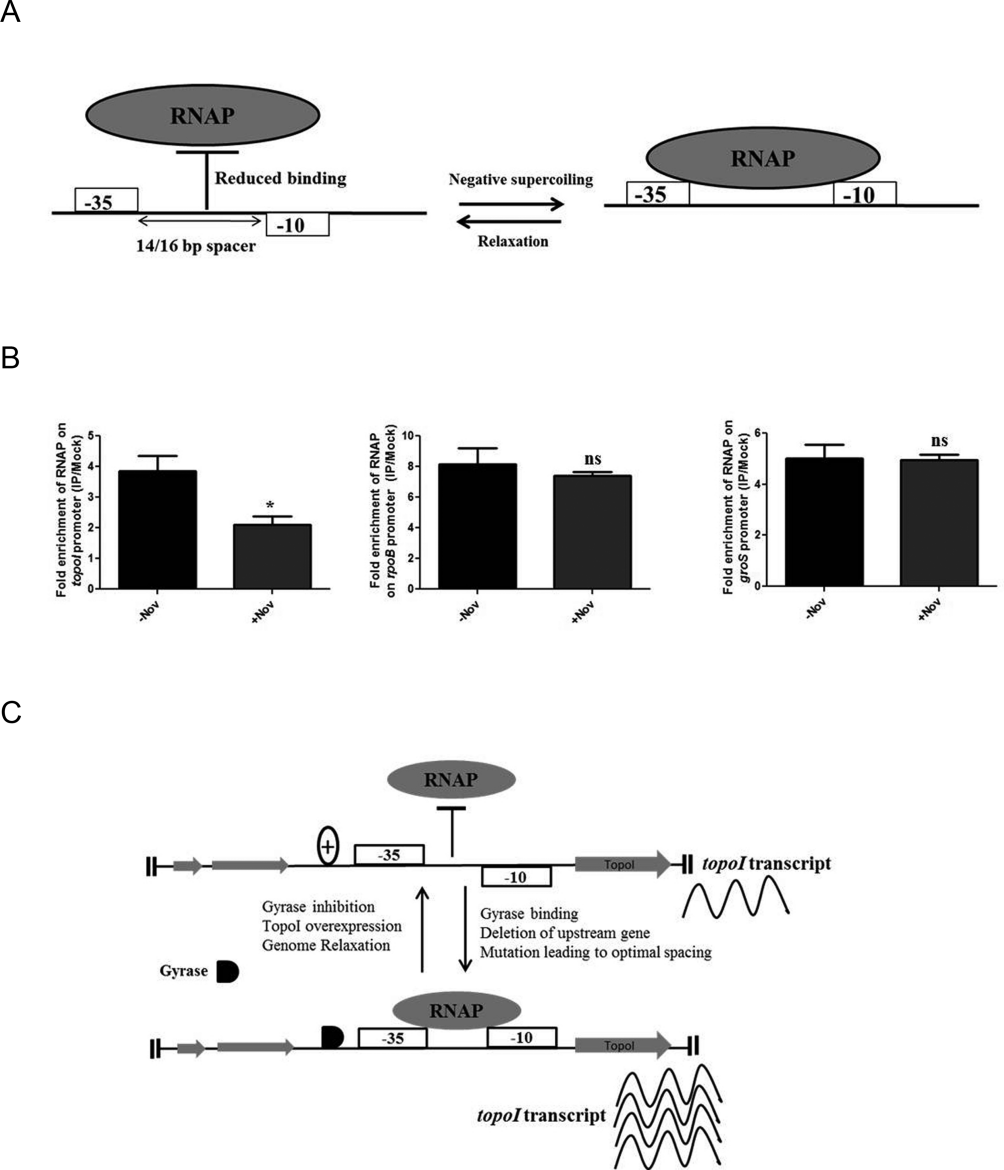
Effect of chromosome relaxation on RNAP binding on TopoI promoter(s). (**A**) Schematic of the experimental design. Negative supercoiling may bring the −35 and −10 elements of *topoI* promoter(s) on the same phase of DNA thus enhancing the RNAP-promoter interaction. (**B**) Exponentially grown *M. smegmatis* cells were treated with novobiocin (100 μg/ml) for 6 h. The treated cells were subjected to ChIP using anti-RpoB antibody. The immunoprecipitated DNA was analyzed for the enrichment of promoter region of various genes by qRT-PCR using primers flanking the promoter region. (**C**) Model representing the SST of TopoI. Sub-optimal spacer length between −10 and −35 elements align them out of phase affecting the optimal RNAP-promoter interaction. Additionally upstream gene transcription affects the DNA topology restricting the *topoI* activity. The negative supercoiling activity of DNA gyrase maintains the optimal topology of the *topoI* promoter aligning the −35 and −10 elements in the appropriate orientation required for the optimal transcription. Conditions leading to the chromosome relaxation reduce the *topoI* expression by altering optimal orientation of −35 and −10 elements. The error bars represent the SD obtained from three independent experiments.*P < 0.01, ns: not significant (*P* > 0.1).

The relative orientation between the −35 and −10 regions can strongly influence the ability of the RNA polymerase (RNAP) to locate and bind to a promoter (44). Supercoiling changes can twist/untwist the DNA and thus can change the orientation of the −35 and −10 elements, potentially affecting the interaction of RNAP with the promoter(s). The requirement of gyrase activity for the active expression of *topoI* promoters indicates that the negative supercoiling may provide an optimal template topology for the efficient binding of RNAP during the transcription initiation. To evaluate the effect of chromosome supercoiling on the binding of RNAP on the *topoI* promoter(s), the ChIP of RNAP was carried out to determine the RNAP occupancy on *topoI* promoter region. The exponential phase cells were treated with novobiocin and subjected to ChIP-qRT PCR analysis. Upon relaxation of the chromosome, the occupancy of RNAP on the *topoI* promoter(s) reduced significantly compared to the untreated cells (Figure 6). In contrast the occupancy of RNAP was not reduced on the promoter region of the supercoiling insensitive *rpoB* and *groS* genes. The occupancy of RNAP on the *topoI* promoter construct harboring optimal spacer length did not vary upon chromosome relaxation (Supplementary Figure S11C). These results imply that negative supercoiling would induce the topological changes in the DNA/promoter thus allowing optimal RNAP binding to the *topoI* promoter(s).

## DISCUSSION

The environmental conditions (24,45,46) tend to perturb the bacterial chromosome supercoiling influencing various cellular processes (39,47) and hence it is necessary to maintain supercoiling homeostasis of the chromosome. Topoisomerases ensure the topological homeostasis by introducing or removing the supercoils into DNA. Studies carried out in *E. coli* indicated the supercoiling sensitive expression of topoisomerases (10,12,14) which serves as a sensor of topological state of the chromosome. Here, we describe the supercoiling sensitive expression of *topoI* in mycobacteria and elucidate the underlying molecular mechanism. The role of high transcriptional activity of an upstream gene and the promoter architecture of *topoI* in the regulation is unraveled. Either ectopic overexpression of TopoI or inhibition of gyrase induced chromosome relaxation ensured that the endogenous TopoI expression is sensitive to the supercoiling status of the chromosome. Notably, in contrast to *E. coli* where overproduction of TopoI affected the plasmid topology marginally, overexpression of *M. smegmatis* TopoI lead to a significant level of relaxation of plasmid DNA. The enhanced relaxation achieved by mycobacterial TopoI could be associated with the high processivity of the mycobacterial TopoI (48). Being a sole relaxase in mycobacteria, it has to sense supercoiling states and respond to maintain the topological homeostasis. The *topoI* promoters were active only in mycobacteria (and not in *E.coli*) suggesting the unique organization of these promoters. The sequence of −35/−10 elements or overall architecture of *topoI* promoters may govern their recognition by mycobacterial transcription machinery but not *E. coli*. The analysis of the promoters after TSS mapping revealed unusual −35 and −10 elements (compared to *E. coli*) in both the mycobacteria which could confer the specificity observed. The species specific activity of mycobacterial promoters is not restricted to the *topoI* promoter (s). Both the *M. smegmatis* and *M. tuberculosis* gyrase promoters were found to be active only in mycobacteria and not in *E. coli* (18,19). The recognition sequence of the mycobacterial *topoI* and DNA gyrase promoters (18,19) were significantly different from the σ70 consensus recognition (49) sequence of *E. coli* which may account for the difference in the expression of these promoters in the two bacterial genera. The importance of promoter architecture on transcription efficiency is well established (50,51). In addition to −10 and −35 elements, the role of discriminator region and spacer sequence between the two elements determines the promoter strength (52–54). Due to the helical nature of the DNA, increase or decrease in spacer length from 17(±1) would affect the orientation of the promoter elements rendering their activation sensitive to supercoiling. The alignment of −35 and −10 elements may contribute to the SST of the mycobacterial *topoI*. Replacement of native *topoI* promoter(s) with the P*ptr* which has a 17 bp spacer did not show supercoiling sensitivity strengthening the hypothesis that the SST is intrinsic to native *topoI* promoters. Due to the helical nature of DNA, the 17 bp spacing between the −35 and −10 elements aligns them in an orientation facilitating the promoter recognition and binding by RNAP (55). Under the situation where the spacing is different, the binding of RNAP may reduce and thus the introduction of supercoiling may facilitate initiation of transcription. ChIP analysis for the occupancy of RNAP on the *topoI* promoter(s) revealed the importance of supercoiling for the optimal binding of RNAP. Upon chromosome relaxation, the RNAP occupancy was decreased which could be a consequence of loss of optimal orientation of −35 and −10 elements upon chromosome relaxation. Studies with the *E. coli tyrT* indicated the role of supercoiling in its regulation. ‘Twist’ model proposed by Wang and Syvnanen suggested the role of suboptimal spacer in conferring the supercoiling sensitivity to various promoters. Based on the model, cold shock response of recA, osmotic shock response of *proU*, genes involved in the stringent response, regulation of histidine operon were predicted to be the supercoiling sensitive as these promoter(s) harbor suboptimal spacer length (44). Moreover, the sub-optimal spacing of *tyrT* promoter element (16 bp) was responsible for supercoiling sensitivity and insertion of a single nucleotide in the spacer rendered it supercoiling insensitive (56). Similarly, in *Helicobacter pylori fla*A having sub-optimal spacer length of 13 bp between the promoter elements, its expression was found to be sensitive to chromosome supercoiling (57). In all these cases, the impact of spacer length on the gene expression seems to be an attribute of promoter-RNAP interaction which needs to be further explored. The out of phase orientation of −35 and −10 elements would require additional factor (58,59) and/or supercoiled induced twisting (44) to bring them in phase allowing the optimal RNAP-promoter interaction as in the case of *proU* (60) and the present study. The negative supercoiling mediated regulatory mechanism of *topoI* deciphered here validates the ‘twist’ model proposed earlier (44).

The *E. coli topoI* promoters also exhibit the suboptimal spacer length (21) but their role has not been addressed in the supercoiling mediated regulation. Instead the role of a topology sensory protein Fis has been implicated in the *topoI* regulation (61). Fis binding on the *topoI* promoter was shown to modulate its activity differentially. Notably, Fis is absent in mycobacteria. In the absence of Fis, mycobacteria could have evolved the operation of suboptimal spacer based direct supercoiling sensitive regulatory mechanism of *topoI* expression.

In addition to the promoter architecture, transcriptional activity of the neighboring gene appears to influence the activity of the *topoI* promoter. The studies with a mutant *leu-500* promoter provided one of the first evidences for the role of DNA topology in promoter activity (62,63). The transcriptional activity of a divergent promoter inserted upstream to the *leu-500* led to the enhanced negative supercoiling resulting in higher expression of *leu-500* (64). Further, the insertion of DNA sequences which are more prone to DNA melting upstream to the promoter enhances the transcriptional activity of the downstream promoter (65). According to the twin-supercoiled domain model of transcription, positive supercoils are generated ahead of the RNAP and negative supercoils upstream (40,41). The positive and negative waves of supercoiling would alter the local DNA topology (66) and thus influence the activity of the promoters residing in the region. In the present study, the deletion of gene upstream to *topoI* resulted in transcriptional inactivation leading to the reduced accumulation of positive supercoils ahead of *topoI* promoter. Hence, the activity of supercoiling sensitive *topoI* promoter was increased. The high occupancy of DNA gyrase at a region upstream to *topoI* implicates its role in the removal of positive supercoils and maintenance of optimal supercoiling around *topoI* regulatory region. Moreover, the negative supercoiling would also facilitate the melting of GC rich recognition and discriminator sequence of *topoI* promoter which is required for the optimal RNAP-promoter interaction (3,67–69). Therefore the inhibition of DNA gyrase by novobiocin would lead to chromosome relaxation resulting in reduced *topoI* transcription (Figure 6C).

Various environments and metabolic states influence the global chromosome supercoiling and thus the activity of supercoiling sensitive promoter(s). In *E. coli* and *Salmonella*, osmotic stress induced chromosome supercoiling resulted in enhanced expression of *proU* required for the adaptation (35). Analysis of the architecture of *proU* promoter revealed the presence of suboptimal spacer (16 bp) which was shown to confer supercoiling mediated osmoregulation of the gene (60). The *topoI* promoter exhibited the alteration in its activity in response to the environmental condition known to affect the supercoiling of the cellular DNA. The osmotic shock is known induce the supercoiling of DNA (35,36,70) and hence it led to the enhanced mycobacterial *topoI* promoter activity. On the other hand, contrary to *E. coli* paradigm high temperature resulted in a relaxation of the DNA and concomitant decrease in the *topoI* promoter activity. The observed discrepancy could be associated with the differences in the physiology and metabolism between the bacterial species. The perturbation of mycobacterial *topoI* promoter activity in response to the environmental cues suggested its role in sensing the environment and generating appropriate response for maintaining cellular homeostasis. Indeed, the chromosome supercoiling modulators *topoI* and DNA gyrase have been implicated in the regulation of various genes including the expression of virulent genes (39,71). The invasive phenotypes of *Shigella flexneri* and *Salmonella* Typhimurium were compromised in a *topoI* mutant (72,73). The hostile environment of the host may alter the chromosome supercoiling of the pathogenic species of mycobacteria which can be sensed by the *topoI* promoter. The subsequent response in the form of altered gene expression may be required for intracellular adaptation.

The alterations in the chromosome supercoiling can impact globally the gene expression profile across various bacterial species (74–76). The expression of TopoI and DNA gyrase is very well co-ordinated, i.e. decrease in gyrase activity would lead to the reduced expression of TopoI (6,15). The mutation of *topoI* in *E. coli* led to the compensatory mutations in the DNA gyrase suggesting the crucial requirement of the balancing activity of the supercoiling and relaxation enzymes (13). In *M. smegmatis*, by overexpressing the TopoI, the expression of DNA gyrase was also increased thus establishing the co-ordinated expression. The strict maintenance of the DNA supercoiling is achieved by the supercoiling sensitive regulatory circuits (RST versus SST) of both the topoisomerases. Another level of regulation of topoisomerase expression in *E. coli* is carried out by Fis which influences the expression of both DNA gyrase as well as *topoI* by modulation of promoter(s) activity (60,77). Mycobacteria lack Fis but HU was found to stimulate the TopoI relaxation activity by direct protein–protein interaction (78). TopoI-HU direct interaction was specific to mycobacteria suggesting the stringent regulation of TopoI activity in mycobacteria which could be associated with its regulatory role inside the cell.

To conclude, this study highlights the importance of supercoiling in the regulation of *topoI* expression in mycobacteria. The autoregulatory mechanism of *topoI* by sensing the alterations in chromosome supercoiling would ensure immediate response in fine tuning the enzyme levels as per cellular requirement. Given the supercoiling sensitivity of both *E. coli* and mycobacterial *topoI* promoters, the autoregulatory mechanism is likely to be conserved across eubacteria. The regulation of *topoI* by the chromosome supercoiling is influenced by a combination of factors such as sequence of promoter elements, spacer length between the elements, neighboring gene expression status and topology modulatory proteins. The contributions of individual factors in the SST of *topoI* need to be dissected by extensive mutational analysis and genetic approaches.

## SUPPLEMENTARY DATA

Supplementary Data are available at NAR Online.

SUPPLEMENTARY DATA
