# Supplementary material for: Autoregulation of topoisomerase I expression by supercoiling sensitive transcription
Source: Nucleic Acids Res. 2015 Oct 22;44(4):1541–52. doi: 10.1093/nar/gkv1088 (PMC4770202; doi:10.1093/nar/gkv1088)
Supplement: SUPPLEMENTARY DATA [file supp_44_4_1541__index.html]

Autoregulation of topoisomerase I expression by supercoiling sensitive transcription — SUPPLEMENTARY DATA 

# Autoregulation of topoisomerase I expression by supercoiling sensitive transcription

## SUPPLEMENTARY DATA

- SUPPLEMENTARY DATA
