# Supplementary material for: Autoregulation of topoisomerase I expression by supercoiling sensitive transcription
Source: Nucleic Acids Res. 2015 Oct 22;44(4):1541–52. doi: 10.1093/nar/gkv1088 (PMC4770202; doi:10.1093/nar/gkv1088)
Supplement: SUPPLEMENTARY DATA [file supp_gkv1088_nar-02363-x-2015-File008.pdf]

(a)

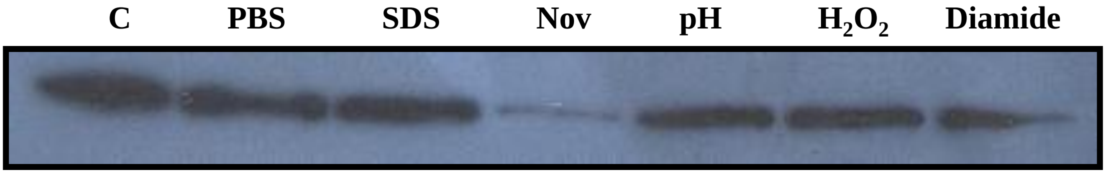

(b)

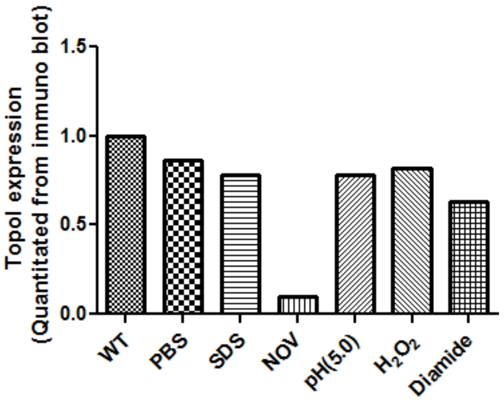

Supplementary Figure S1. TopoI expression profile under different stress conditions (a) *Msm* cultures were grown to O.D $_{600nm}$= 0.5-0.6. The cultures were treated with different reagents to give stress for 3 hrs and subsequently the cells were lysed. The 20 µg of cell lysate were loaded on the gel and subjected to immunoblot analysis (b) Quantitation of TopoI expression.

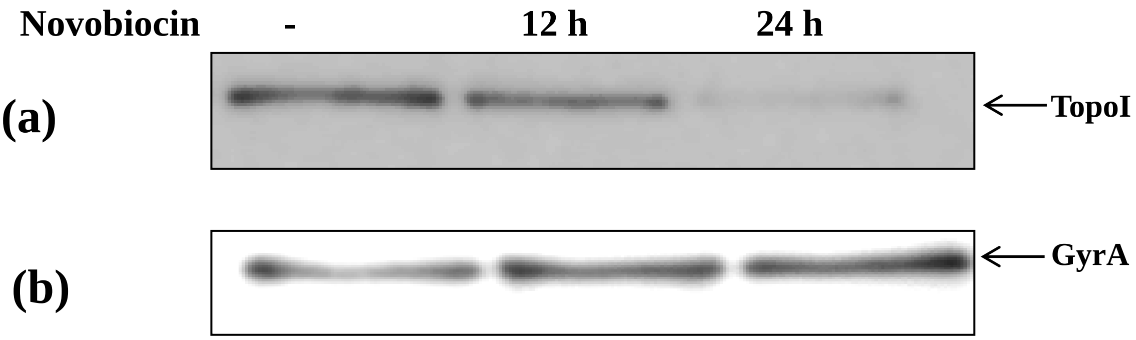

Supplementary Figure S2. Analysis of TopoI and Gyrase expression in *Mtb*. Exponential phase cultures of *Mtb* cells were exposed to novobiocin (100 µg/ml) for indicated period of time. (a) Expression analysis of *Mtb* TopoI (b) Expression analysis of *Mtb* GyrA.

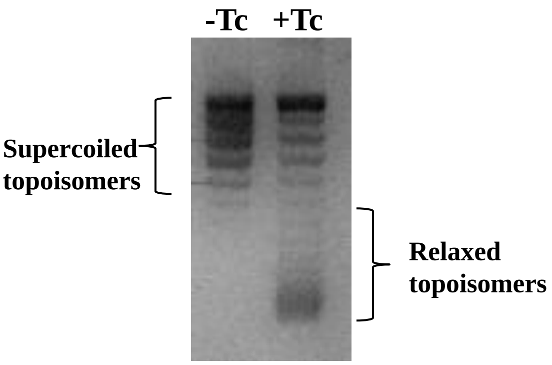

Supplementary Figure S3. Plasmid relaxation by TopoI overexpression in *M. smegmatis*. The exponential phase cultures of *M. smegmatis* harboring the pMIND-topoI plasmid were treated with tetracyline (25 ng/ml) for 6 h and processed for plasmid isolation as described previously (Ahmed W et al., (2015) *Microbiology*). The purified plasmid (500 ng) topoisomers were resolved on the choloroquine (15 µg/ml) agarose gel. At this concentration of chloroquine plasmid topoisomers with higher linking numbers (relaxed) run faster than the ones with lower linking number (negatively supercoiled).

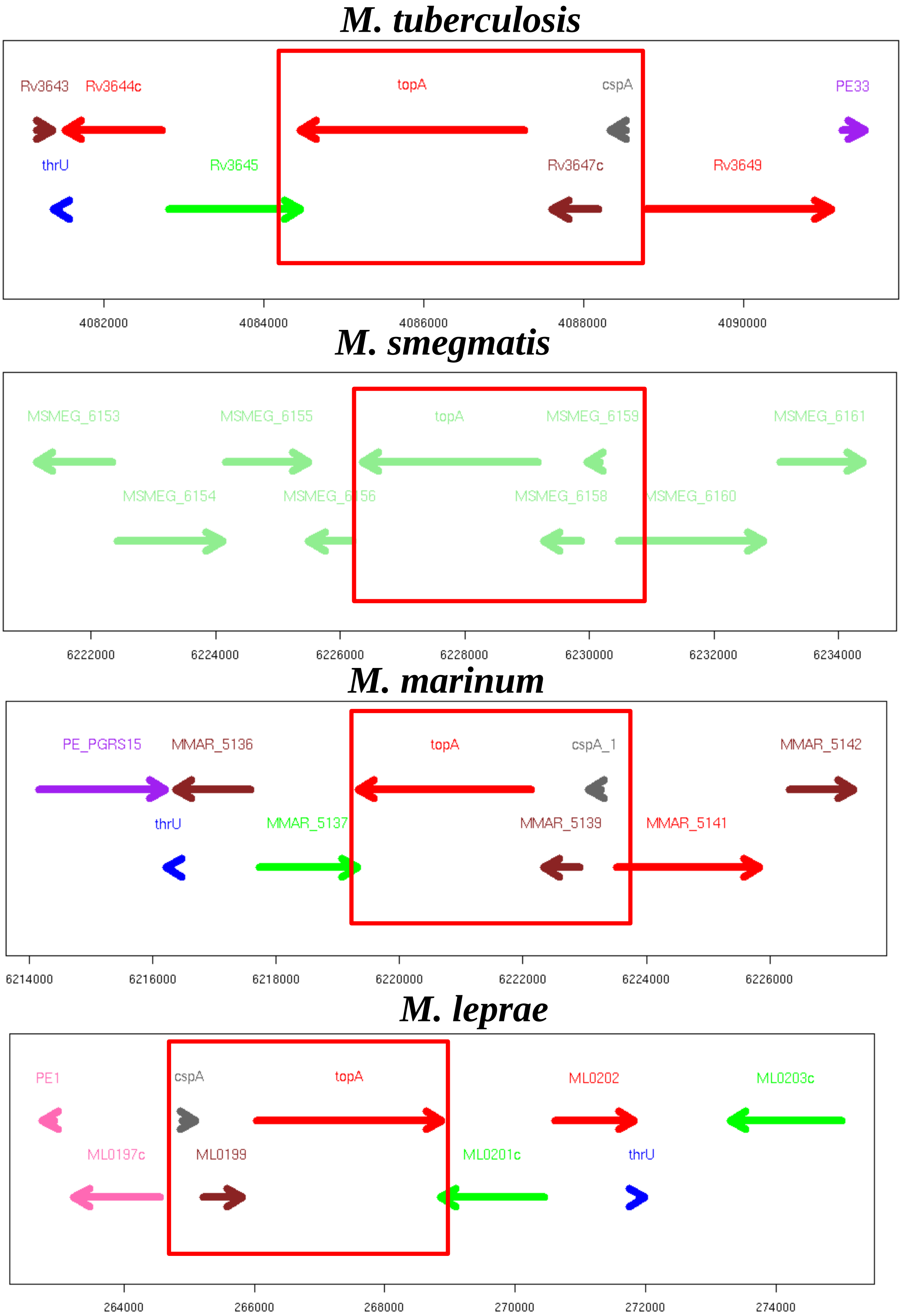

Supplementary Figure S4. Conserved genomic arrangement of *topoI* gene and upstream neighboring genes across different mycobacterial species. The data was obtained from the mycobrowser (http://mycobrowser.epfl.ch/).

(a)

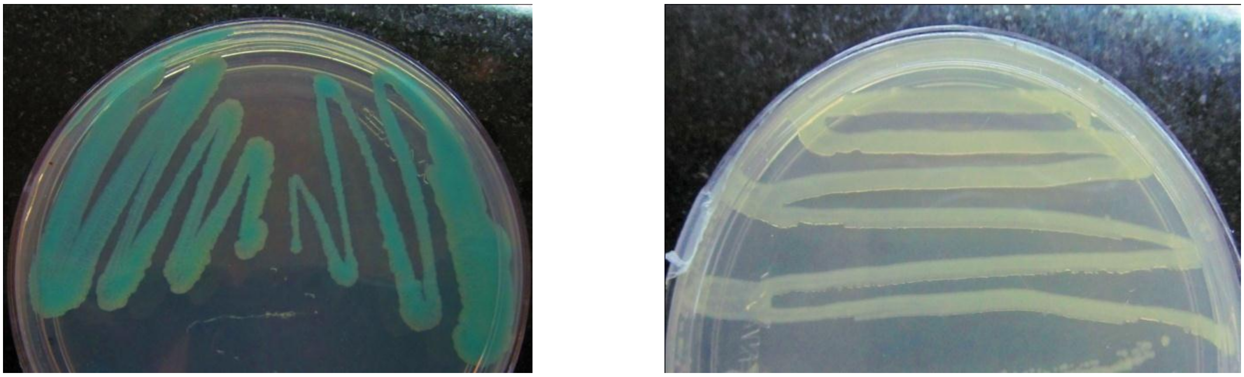

M. smegmatis ***E. coli***

(b)

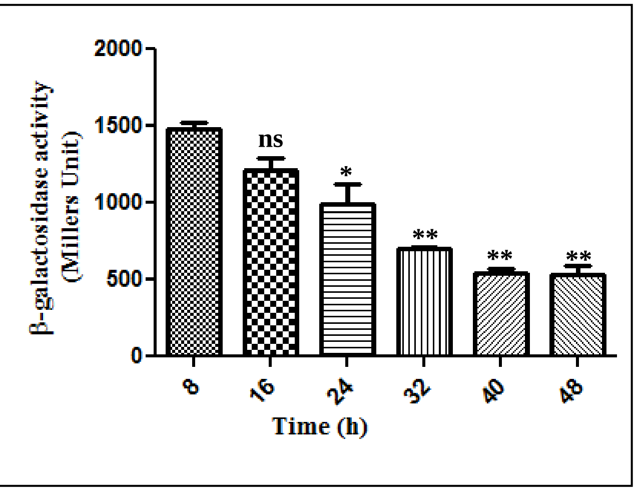

(c)

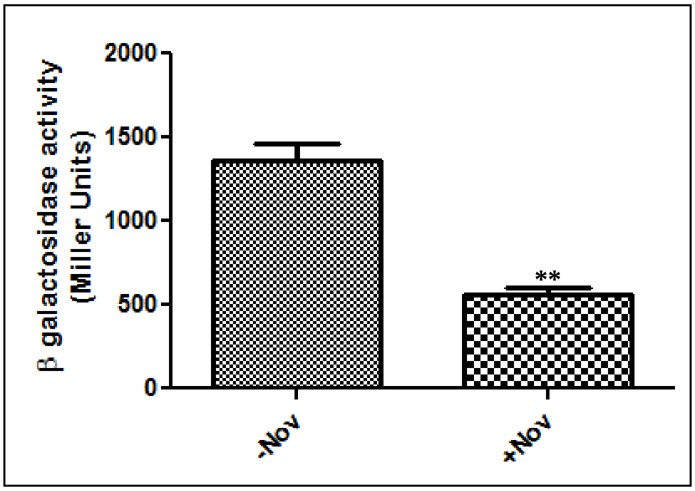

Supplementary Figure S5. *topoI* promoter activity. 1.5 kb upstream region of *topoI* gene was cloned into promoterless pSD5B vector upstream to the β-galactosidase reporter gene. The resulting construct was introduced into *E. coli* as well as *M. smegmatis*. (a) Detection of promoter activity in *M. smegmatis* and *E. coli*. The exponential phase cultures were streaked on the agar plates containing the X-gal (40 µg/ml) and Kanamycin (25 µg/ml) and incubated for 48 h (*M. smegmatis*) and 12 h (*E. coli*). The appearance of blue color colonies is the indication of β-galactosidase expression. (b) β-galactosidase assays carried out to determine the *M. smegmatis topoI* promoter activity under different growth phase. (c) Determination of promoter activity upon gyrase inhibition. Exponential phase culture of *M. smegmatis* was treated with novobiocin (100 µg/ml) for 6 h and β-galactosidase activity was determined. The error bars represent the SD obtained from three independent experiments. *P<0.01, **P<0.001, ns: not significant (P>0.1).

(a)

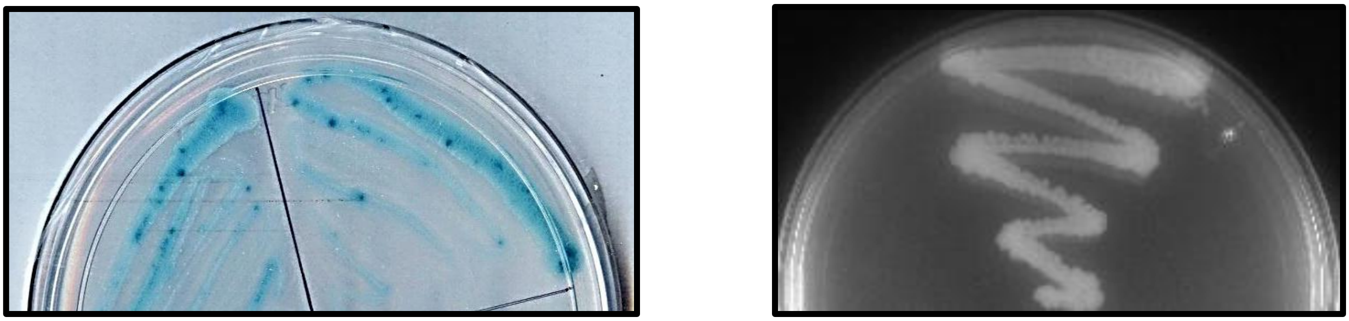

M. smegmatis ***E. coli***

(b)

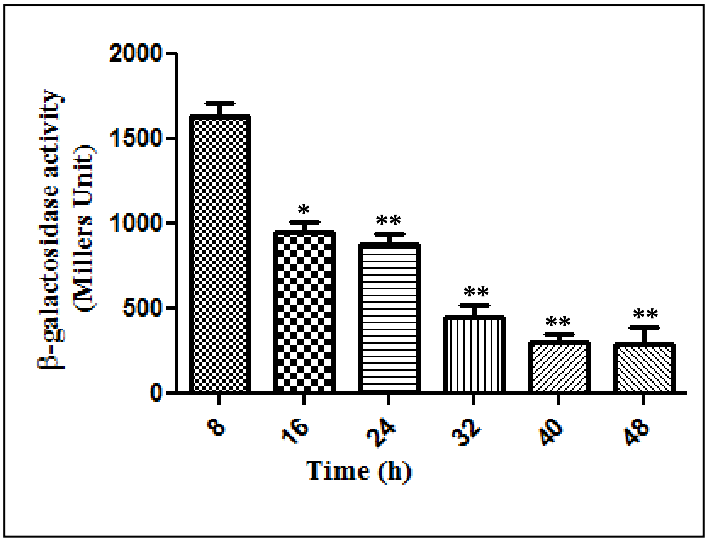

(c)

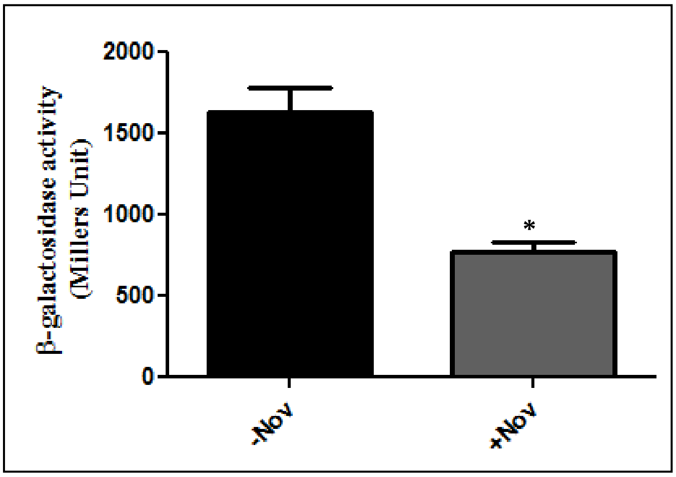

Supplementary Figure S6. *Mtb topoI* promoter activity. (a) *Mtb topoI* promoter activity in *E. coli* and *Msm* (b) The pSD5B constructs with the MttopoI promoter region were electroporated into *Msm* cells and its activity were determined by β-galactosidase assay (b) Under different growth phases (c) Exponential phase culture was treated with novobiocin (100 µg/ml) for 6 h and β-galactosidase activity was determined. Error bars represent the SD obtained in three independent experiments. *P<0.01, **P<0.001

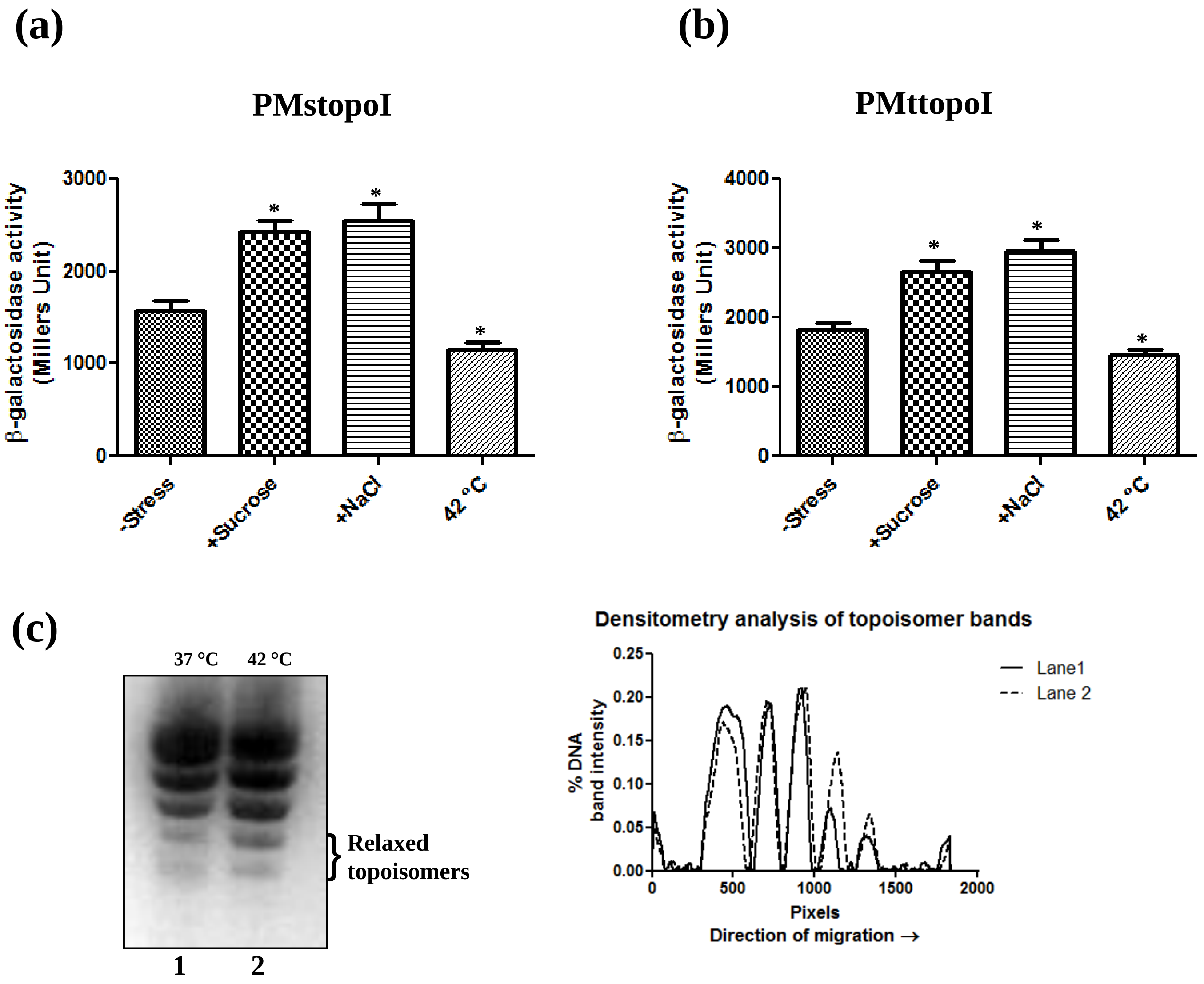

Supplementary Figure S7. Mycobacterial *topoI* promoter activity during different stress conditions. The respective reporter plasmids with PMttopoI or PMstopoI were electroporated in *Msm* cell. The exponential phase cells were exposed to different stress conditions followed by determination of promoter activity. All the stresses were induced for 3 h. For the osmotic stress, 0.5 M NaCl or 0.5 M sucrose was used, Temperature stress was induced by shifting the culture to 42 °C for 3 h. (a) TopoI promoter region from *Msm* (b) TopoI promoter region from *Mtb*. (c) The exponential phase *M. smegmatis* cells harboring pMV261 plasmid were shifted to 37°C and 42C° for 6 h. The cultures were harvested and processed for the plasmid isolation as described previously (Ahmed W et al., (2015), *Microbiology*). The isolated plasmids were analyzed on chloroquine (15 µg/ml)-agarose gel . At this concentration of chloroquine plasmid topoisomers with higher linking number moves faster than those with lower linking number. The topoisomer distribution density was determined by rolling ball method. Error bars represent the SD obtained in two independent experiments. P<0.01.

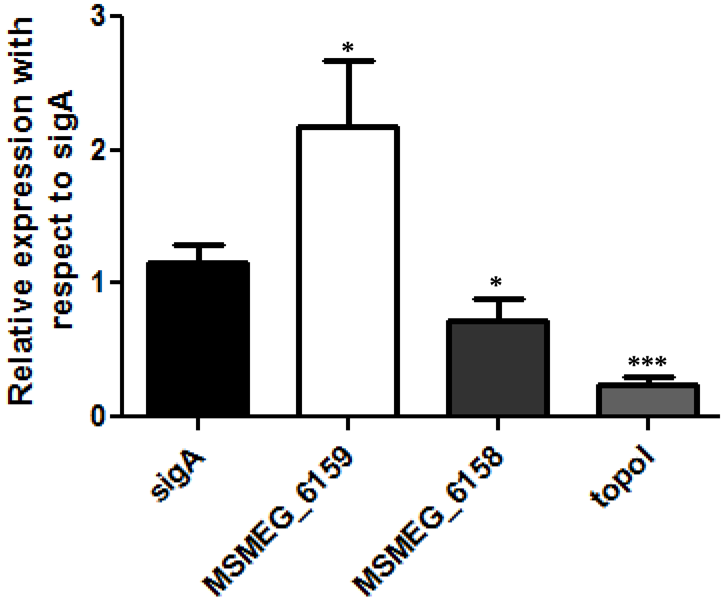

Supplementary Figure S8. Expression analysis of *topoI* upstream genes. RNA was isolated from the exponential phase cells and subjected to cDNA synthesis using gene specific primers. Semi-quantitaive RT PCR was carried out to determine the copy number of the transcripts in 20 ng of cDNA. The relative abundance of the gene transcripts was plotted with respect to the sigA expression. Error bars represent the SD obtained in three independent experiments. * P<0.01, *** P<0.0001.

(a)

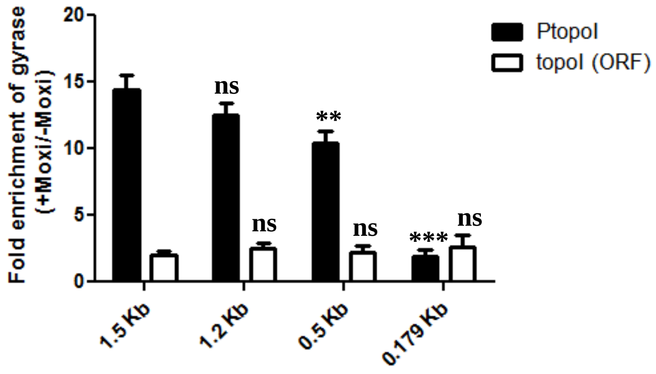

(b)

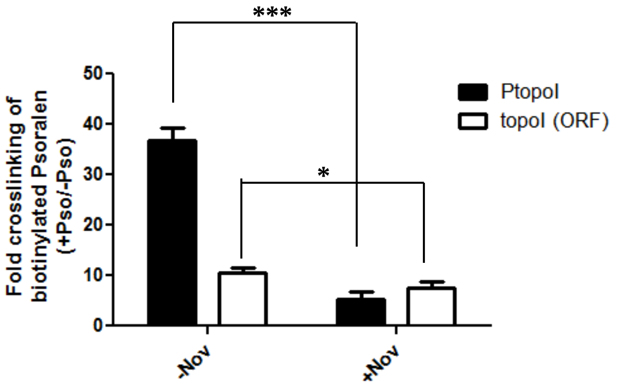

Supplementary Figure S9. Gyrase is recruited on *topoI* upstream region to introduce negative supercoiling (a) Determination of gyrase trapping on *topoI* promoter region by moxifloxacin. The exponential cells harboring the plasmid constructs were treated with 5X MIC of moxifloxacin (moxi) for 3 h to trap the gyrase-DNA cleavage complex. Following the trapping, ChIP was carried out using the GyrA specific antibody. The representative enrichment values were obtained by normalization of the data with the vector control. **(b) Monitoring the effect of gyrase inhibition on supercoiling at *topoI* upstream region.** Novobiocin treated and untreated cultures (15 ml) were incubated with the 2 ml of biotinylated Psoralen (Pso/100 µg/ml)) and crosslinking of Psoralen was induced by exposing the cells to 365 nm light for 20 min (Naughton C et al., (2013) *Nat. Struct. Mol. Biol*.). Following the crosslinking cells were resuspended in the IP buffer and subjected to ChIP using the streptavidin beads. The IP DNA was subjected to qRT PCR analysis to monitor the binding of psoralen to DNA which is an indicative of negative supercoiling. The *topoI* promoter specific and ORF specific primers were used to determine the gyrase binding. PtopoI and *topoI* (ORF) represents the occupancy of DNA gyrase on *topoI* upstream region and ORF respectively. The error bars represents the SD obtained from three independent experiments.*P<0.01, ***P<0.0001.

(a)

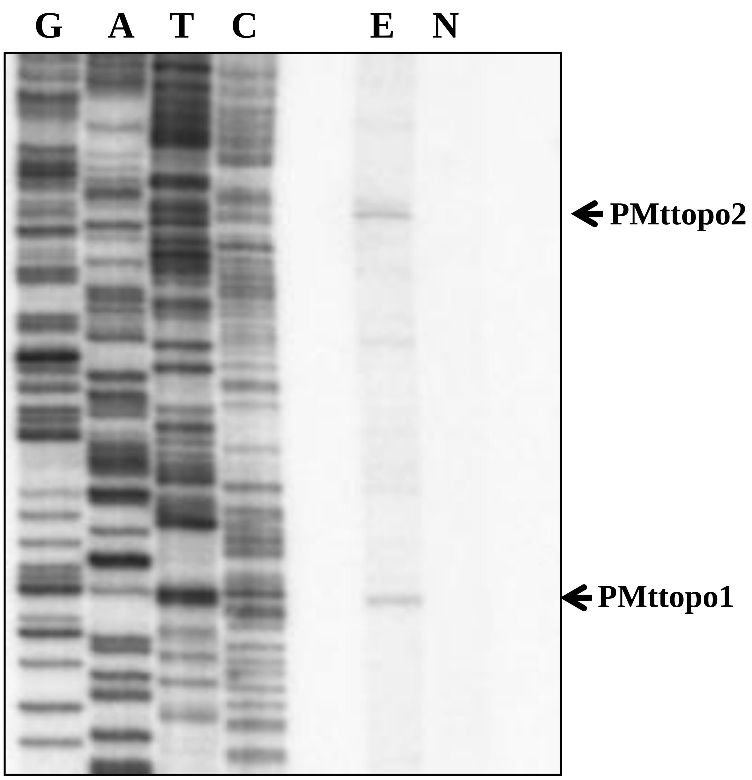

(b)

TSS	Nucleotide	Length of 5'UTR
MtPtopo1	T	123
MtPtopo2	A	166

(c)

-35 -10 -35

tgagcggcccGTTTGAgcctttggtgtgggTAATCTgtttgcAgccggttTGCGCA

PMttopo2

PMttopo1

ggcccgccctagagTGCGAGattgtcagtTgcccacaggcgagggaacgccggcgtaccg

-10

gaattcacctgggattcggcagtcggccgcgtcctctacctaccggggcggtctcgataggggccg

ggataagagatggagcgtgggcgcag

Supplementary Figure S10. Identification of Transcription Start Sites of topoI gene from *Mtb* (a) Primer extension analysis was carried out to map the TSS upstream of *topoI* gene as described in materials and methods. The primer extension product corresponding to the transcription start site for each promoter is indicated. E; exponential phase culture with out novobiocin treatment, N; Exponential phase culture treated novobiocin (100 μg/ml) for 12 h (b) Table representing the 5' UTR length and first nucleotide of the each transcripts. (c) Upstream putative promoter elements of the *M. tuberculosis topoI* .

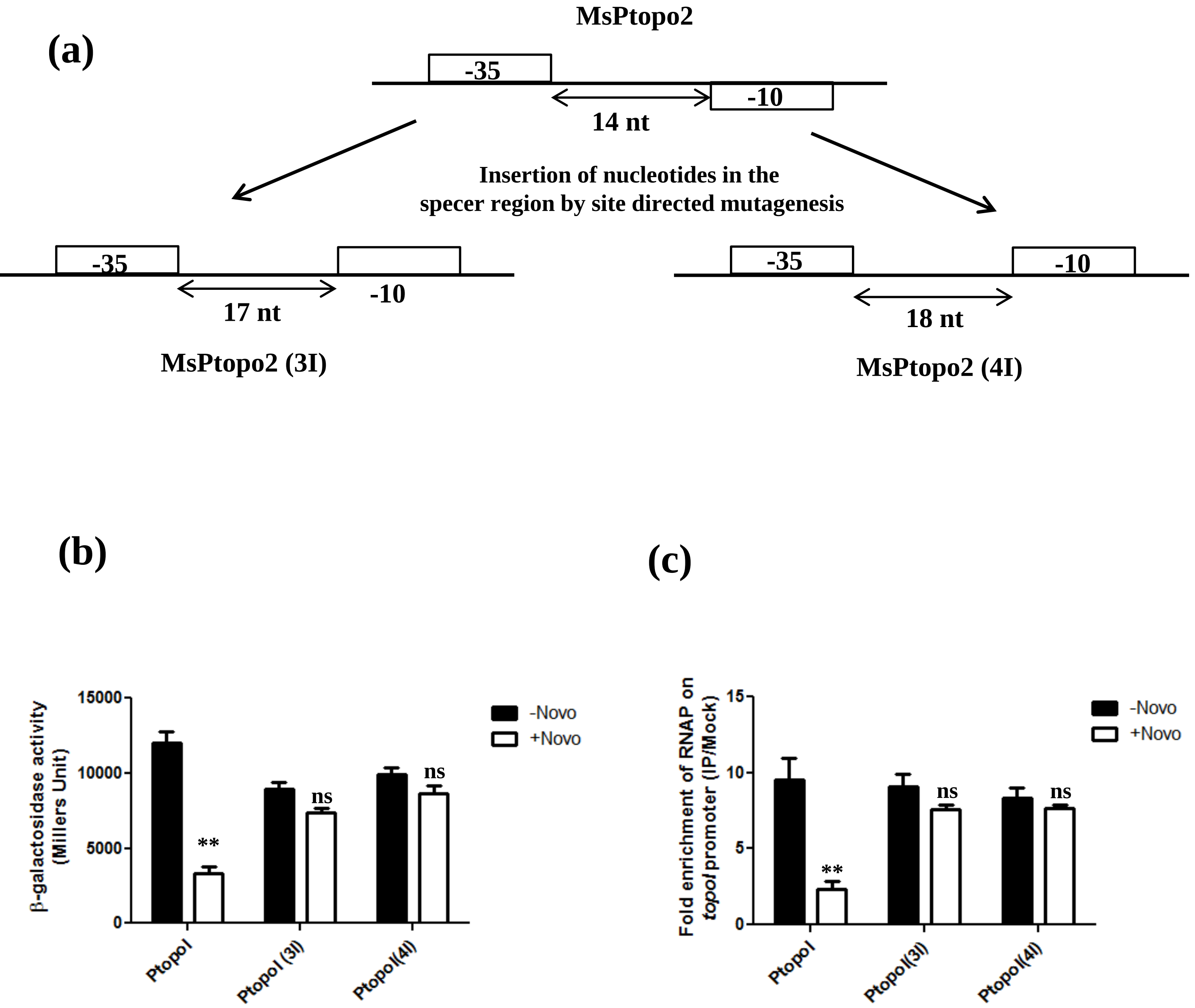

Supplementary Figure S11. Effect of the insertion of nucleotides in the PMstopoI spacer region on the promoter activity in response to the genome relaxation. (a) 530 bp *topoI* promoter fragment cloned into the pSD5B was used a template to introduce the nucleotides in the spacer region of the major promoter MsPtopo2 by site directed insertional mutagenesis. PtopoI (3I): 3 nucleotides (GAT) were included in the in spacer region of *topoI* promoter. PtopoI (4I): 4 nucleotides (GATC) were introduced in the spacer region of *topoI* promoter. **(b)** pSD5B constructs with the various MstopoI promoters (native and recombinant) regions were electroporated into *Msm*. The exponential phase cells harboring the constructs were treated with novobiocin (100 µg/ml) for 6 h and the promoters activity was determined by β-galactosidase assay. (c) The binding of RNAP on the promoter region of WT and recombinant plasmid constructs was determined by the ChIP qRT PCR. The enrichment of RNAP obtained was normalized with the enrichment values obtained from the strain containing only pSD5B (vector control). The data was plotted and analyzed by Graphpad Prism 5.0. Error bars represent the SD obtained in three independent experiment. ****** P< 0.001, ns : not significant.
